# Value of Contrast-Enhanced Ultrasound in Partially Cystic Papillary Thyroid Carcinomas

**DOI:** 10.3389/fendo.2021.783670

**Published:** 2021-12-08

**Authors:** Fengkai Fang, Yi Gong, Liyan Liao, Fei Ye, Zhongkun Zuo, Zhang Qi, Xiaodu Li, Chengcheng Niu

**Affiliations:** ^1^ Department of Ultrasound Diagnosis, The Second Xiangya Hospital, Central South University, Changsha, China; ^2^ Department of Thyroid Surgery, The Second Xiangya Hospital, Central South University, Changsha, China; ^3^ Department of Pathology, The Second Xiangya Hospital, Central South University, Changsha, China

**Keywords:** thyroid carcinomas, partially cystic thyroid nodules, thyroid ultrasonography, partially cystic papillary thyroid carcinomas (PCPTCs), contrast-enhanced ultrasound (CEUS)

## Abstract

Partially cystic papillary thyroid carcinomas (PCPTCs) are rarely reported papillary thyroid carcinomas (PTCs) and are usually misdiagnosed as benign nodules. The objective of this study was to provide the various sonographic characteristics of partially cystic thyroid nodules for differentiation between malignant and benign nodules, including those for conventional ultrasound (US) and contrast-enhanced ultrasound (CEUS). Twenty-three PCPTC patients and 37 nodular goiter patients were enrolled in this study. We evaluated the size, cystic percentage, solid echogenicity, calcification, vascularity, and CEUS parameters for each nodule. The final diagnosis of all patients was confirmed *via* surgery. Univariate analysis demonstrated that compared with benign nodular goiters, PCPTCs more frequently presented with calcification, hypoechogenicity of the solid part, hypoenhancement, heterogeneous enhancement, centrifugal perfusion, peak intensity index <1, time to peak index ≥1, and area under the curve index <1 on preoperative US and CEUS. Binary logistic regression analysis demonstrated that heterogeneous enhancement, centrifugal perfusion, and peak intensity index <1 are independent CEUS characteristics related to malignant PCPTCs and can be used for their differentiation from benign nodular goiters (all p < 0.05). Our study indicated that preoperative CEUS characteristics may serve as a useful tool to distinguish malignant PCPTCs from benign thyroid nodules.

## Introduction

Thyroid carcinomas have a mostly solid composition, but those with predominant cystic changes (>50% of the nodule) can be observed in 2.5%–6.0% of all thyroid carcinoma cases (1, 2). In a prospective study, 213 partially cystic thyroid nodules in 196 patients who had consecutively undergone prospective sonographic diagnosis and ultrasonography-guided fine-needle aspiration biopsy (US-FNAB) were included, and the rate of malignancy for partially cystic thyroid nodules was 5.2% (3). Thyroid nodules can be classified as cystic or almost completely cystic, spongiform, mixed cystic and solid, solid, or almost completely solid according to their composition as ascertained by ultrasonography (4). However, to our knowledge, there are few studies that have investigated sonographic features as predictors for the diagnosis of malignant partially cystic thyroid nodules.

US-FNAB is the preferred method for the preoperative diagnosis of benign and malignant thyroid lesions. Current guidelines consider a size ≥1 cm (in nodules with high suspicion), 1.5–2.0 cm (in nodules with any suspicious US features), or 2.0 cm (in nodules without any suspicious US features) as a criterion for US-FNAB, regardless of the cystic portion (5). However, US-FNAB has a high rate of nondiagnostic and false-negative results for the diagnosis of partially cystic thyroid nodules (6).

Contrast-enhanced ultrasound (CEUS), as a relatively novel ultrasound (US) technique, has great significance value in the diagnosis of collapsing benign cystic or predominantly cystic thyroid nodules when combined with clinical history according to the 2020 Chinese guidelines for ultrasound malignancy risk stratification of thyroid nodules (7, 8). However, very few published studies have reported the use of CEUS for predominant cystic thyroid carcinomas. To our knowledge, this is the first article describing the CEUS features and corresponding histopathology of PCPTCs. Hence, the aim of the present study was to provide CEUS characteristics for PCPTCs in order to distinguish them from benign nodules.

## Materials and Methods

### Patients

The study was approved by the Ethical Committee of the Second Xiangya Hospital of Central South University in China and was performed in accordance with the Declaration of Helsinki for human studies. The requirement of informed consent from human subjects is sometimes waived by institutional review boards (IRBs) for protocols that include a retrospective review of images acquired for clinical diagnostic purposes. From June 2017 to August 2021, 27 partially cystic thyroid carcinoma patients who received conventional US and CEUS examinations were retrospectively enrolled in this case-control study. The inclusion criteria were as follows: 1) patients with mixed echoic thyroid nodules that were confirmed as PTCs with partial cystic degeneration by pathologic examinations after surgery; 2) no invasive procedure such as thyroid surgery or FNA was previously performed. Four patients were excluded because they had different types of thyroid cancers: two medullary thyroid carcinomas and two follicular carcinomas. For patients with multifocal PTCs, only the largest was selected. Finally, 23 patients with 23 PCPTCs were included in this study. In addition, from August 2017 to August 2021, 37 patients with 37 mixed echoic thyroid nodules who received conventional US and CEUS examinations were recruited for this study as a control group. The inclusion criteria were as follows: 1) patients with mixed echoic thyroid nodules that were confirmed to be nodular goiters by pathologic examination after surgery; 2) no invasive procedure such as thyroid surgery or FNA was previously performed. For patients with multifocal thyroid nodules, only the largest was selected. Finally, 37 patients with 37 nodular goiters were included in this study. Ultimately, 60 mixed echoic thyroid nodules in 60 patients were enrolled in the study.

### Conventional US

A Siemens Acuson S3000 US scanner (Siemens Medical Solutions, Mountain View, CA, USA) equipped with 9L4 (4–9 MHz) and 18L6 (6–18 MHz) linear array transducers was used for conventional US, and a 9L4 linear array transducer was used for CEUS. All examinations were performed by the same operator with 15 years of experience in thyroid ultrasound diagnosis and 10 years of experience in performing CEUS to prevent bias from different operators and to ensure optimized image quality. All selected thyroid nodules were evaluated by conventional B-mode US and color-Doppler US for the following US features: size (the largest diameter), cystic percentage (≥50% or <50%), solid part echogenicity (hypoechogenicity or isoechogenicity), calcification (present or absent), and internal vascularity (present or absent).

The nodules were classified according to the Thyroid Imaging Reporting and Data System (TI-RADS) proposed by Kwak et al. (9). According to that classification, five US suspicious features (solid component, hypoechogenicity or marked hypoechogenicity, microlobulated or irregular margins, taller-than-wide shape, and presence of microcalcifications) were applied to categorize the thyroid nodules: TI-RADS score 3 (no suspicious US features), 4a (one suspicious US feature), 4b (two suspicious US features), 4c (three or four suspicious US features), and 5 (five suspicious US features). In this study, hypoechogenicity was applied for the solid part of these mixed echoic thyroid nodules.

### CEUS and Analysis

CEUS was performed using contrast pulsed sequencing (CPS) technology [mechanical index (MI) = 0.07]. SonoVue (Bracco, Italy) was injected intravenously as a bolus of 3.0 ml *via* a 20-gauge antecubital vein cannula, followed by a saline flush of 5 ml, with the timer started simultaneously. Thyroid nodule imaging lasted at least 60 s. The CEUS videos were digitally recorded and analyzed with CEUS software (Contrast Dynamics, Mountain View, CA, USA). Time-intensity curves (TICs) of the thyroid within selected regions of interest (ROIs) were acquired, and the contrast enhancement features of thyroid nodules were applied according to our previous study (10). After comparison with the surrounding thyroid parenchymal enhancement, the contrast enhancement features were classified as follows: enhancement type (hyperenhancement, isoenhancement, hypoenhancement), perfusion pattern (centripetal perfusion, the perfusion of microbubbles from the periphery to the center of nodule; centrifugal perfusion, the perfusion of microbubbles from the center to the periphery of nodule), enhancement uniformity (homogeneous, the microbubbles were evenly distributed; heterogeneous, the microbubbles were unevenly distributed), peak intensity (PI; expressed as a percentage), time to peak (TP; expressed in seconds), and area under the curve (AUC; expressed in percentage by seconds). The PI, TP, and AUC of the nodules are reported as indices by the ratio of the ROI of the nodules to the ROI of the thyroid parenchymal tissue. PI index represents the ratio of the quantitative values of peak intensity of the nodule to the quantitative values of thyroid parenchymal tissue. If the average quantitative values of nodule were higher or equal to that of the thyroid parenchymal tissue, the PI index was expressed as ≥1; if this was not the case, the PI index was expressed as <1. Similarly, TP index ≥1 meant that the time to peak of the nodule was slower or equal to that of the thyroid parenchymal tissue; AUC index ≥1 meant that the area under the curve of the nodule was higher or equal to that of the thyroid parenchymal tissue.

### Reference Standard

FNA Bethesda cytology (BC) diagnoses were divided into six categories according to the Bethesda System (5). The histopathological results after surgery were used as the reference standard for the final diagnosis of PTCs or nodular goiters.

### Statistical Analysis

The statistical analysis was performed with SPSS version 21.0 software (SPSS, Chicago, IL, USA). Continuous data are presented as the mean and standard deviation (SD) and compared by the independent t-test. Categorical data were presented as percentages and analyzed by the chi-square test. Binary logistic regression was used to assess significant CEUS features and their independent association with malignant partially cystic thyroid nodules. The sensitivity, specificity, accuracy, positive predictive value (PPV), and negative predictive value (NPV) of TI-RADS for differentiation between benign and malignant thyroid nodules were calculated. A statistically significant difference was determined when p < 0.05.

## Results

A total of 60 patients with 60 partially cystic thyroid nodules (23 malignant and 37 benign) were included in the analysis. For malignant thyroid nodules, the FNA cytology diagnoses for 23 nodules were as follows: one (4.3%) nodule was BC 3 (atypia or follicular lesion of undetermined significance), three (13.1%) nodules were BC 4 (follicular neoplasm or suspicious for a follicular neoplasm), seven (30.4%) nodules were BC 5 (suspicious for malignancy), and 12 (52.2%) nodules were BC 6 (malignant). All nodules were confirmed as PTC by pathologic examinations after surgery. For benign thyroid nodules, 16 patients had malignant-looking thyroid nodules on the other thyroid lobe, and FNA cytology was carried out for the malignant thyroid nodules. Thus, 16 patients with PTCs on the other thyroid lobe were confirmed by pathologic examinations after total thyroidectomy; 16 benign thyroid nodules on this thyroid lobe were also confirmed by histopathology. The FNA cytology diagnoses for the other 21 nodules were as follows: six (28.6%) nodules were BC 3, and 15 (71.4%) were BC 2 (benign).

The clinical characteristics of the patients are outlined in **Table 1**. The average ages of PCPTC patients and nodular goiter patients were 41.74 ± 9.99 years (range: 29–63 years) and 51.92 ± 9.55 years (range: 25–68 years), respectively, and PCPTC patients were younger than nodular goiter patients in this study (p < 0.05). Male patients constituted 26.1% of PCPTC patients and 5.4% of nodular goiter patients; thus, female patients accounted for a larger proportion of nodular goiters than PCPTCs (p < 0.05). Thirteen (56.5%) PCPTC patients and 31 (83.8%) nodular goiter patients had multifocal thyroid nodules, and nodular goiter patients had many more thyroid nodules per patient than PCPTC patients in this study (p < 0.05).

**Table 1:** Clinical characteristics of the PCPTCs and nodular goiters.

Characteristics	PCPTCs (n = 23)	Nodular goiter (n = 37)	p Value
Age (years)	41.74 ± 9.99	51.92 ± 9.55	0.000*
Male sex	6 (26.1)	2 (5.4)	0.045*
Multifocality	13 (56.5)	31 (83.8)	0.020*

*p < 0.05 was considered a significant difference.

PTPTC, partially cystic papillary thyroid carcinoma.

**Figure 1:**
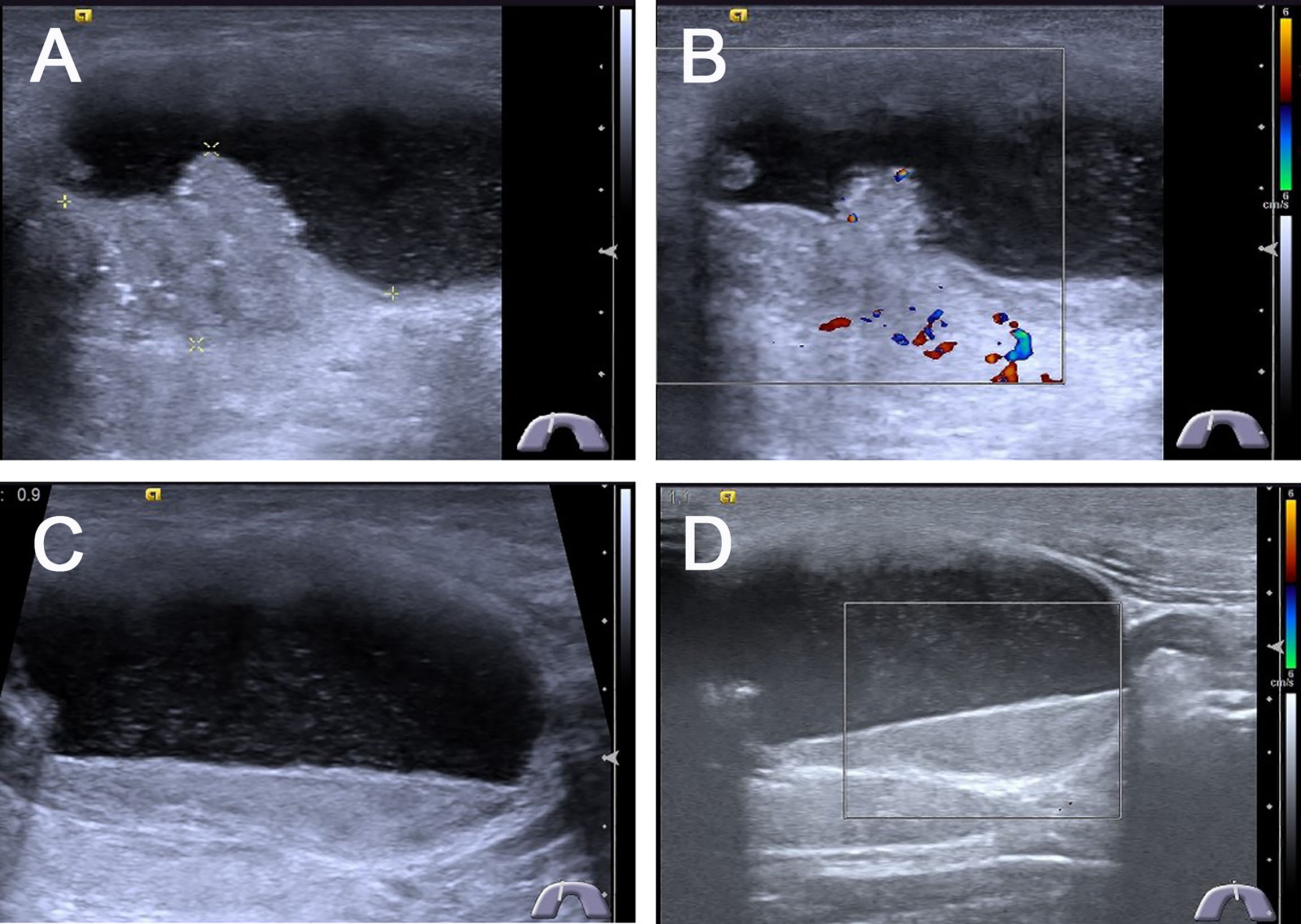
Conventional ultrasonography images of predominantly cystic papillary thyroid carcinoma. **(A)** Longitudinal gray-scale sonography revealed a predominantly cystic 4.2 × 2.4 × 2.7 cm^3^ thyroid nodule with a little solid portion abutted on the side of the cyst wall in the thyroid isthmus, the cystic portion was more than 90% of the thyroid node. **(B)** CDFI showed poor blood flow signals in the solid portion of the thyroid nodule. **(C)** Most portions of this thyroid node were cystic with a lot of mobile silt-like isoechoic substance. **(D)** CDFI showed no blood flow signals in the silt-like isoechoic substance.

**Figure 2:**
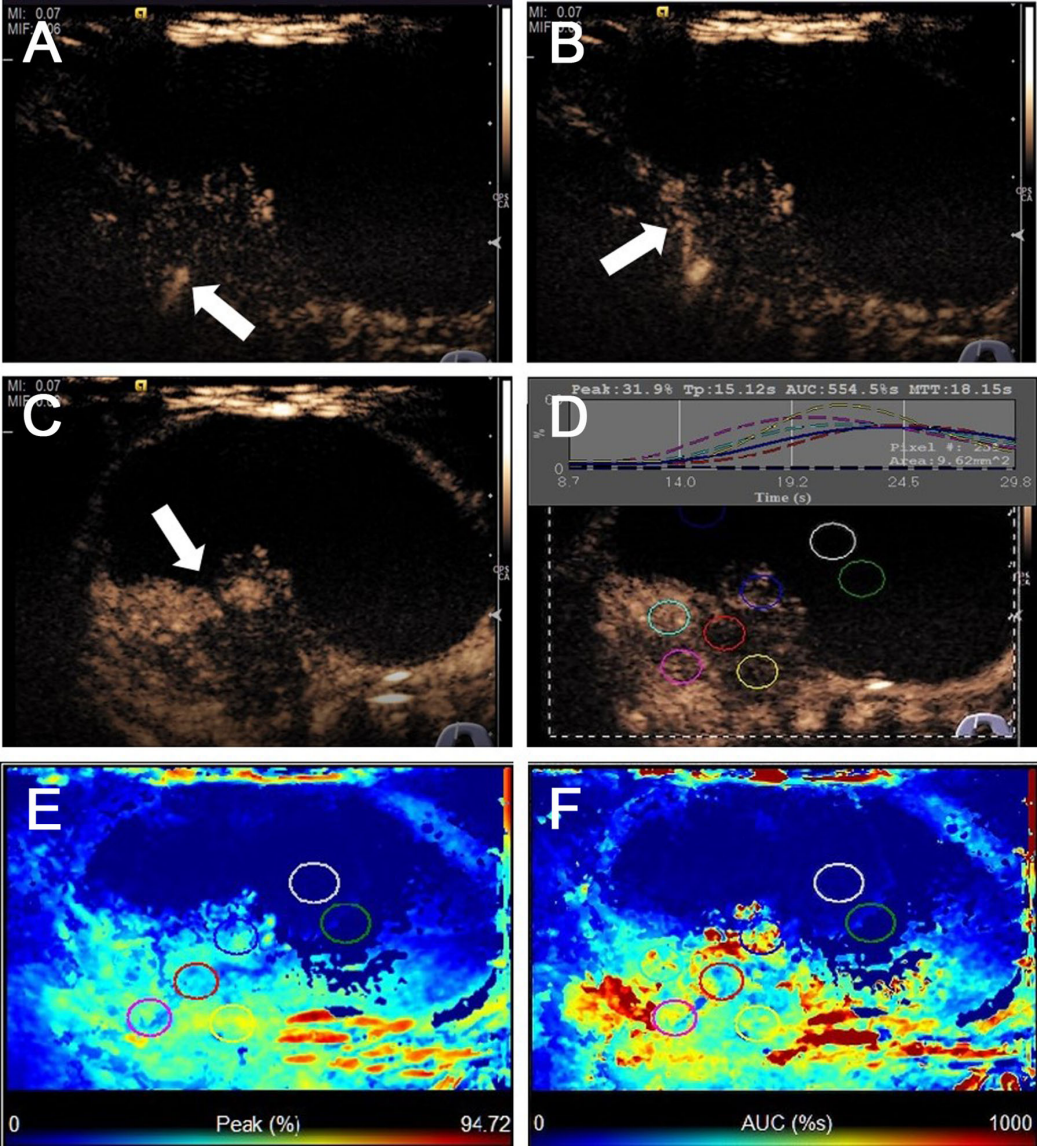
CEUS images of predominantly cystic papillary thyroid carcinoma. **(A)** CEUS image showed a slight enhancement from the bottom of the solid portion at 9 s. **(B)** The enhancement from the bottom to the periphery of the solid portion at 15 s. **(C)** All the solid portions of this nodule heterogeneously enhanced and reached its peak [time to peak (TTP)] at 23 s. **(D)** TICs displayed the wash-in time of 9 s, TTP of 15 s, PI of 31.9%, and AUC of 554.5% s for the solid portion of this thyroid nodule, and the cystic portion of the nodule has no enhancement. **(E)** The parametric color map showed that the solid portion was almost a majority of green with a little blue, the cystic portion was totally blue, which indicated that the PIs for the center of the solid portion was almost equal to those of the periphery of the solid portion. **(F)** The parametric color map showed the solid portion was heterogeneous with a mixture of green, yellow, and red, and the cystic portion was totally blue, which indicated that the AUC for the center of the solid portion was lower than those of the periphery of the solid portion.

The US characteristics of partially cystic thyroid nodules are outlined in **Table 2**. The mean diameters were 29.35 ± 12.21 mm (range: 9–51 mm) for malignant partially cystic thyroid nodules and 32.97 ± 14.39 mm (range: 10–68 mm) for benign partially cystic thyroid nodules. In the malignant thyroid nodule group, six (26.1%) nodules had cystic percentage greater than 50% (**Figure 1**), 21 (91.3%) nodules had calcifications (**Figure 1**), 18 (78.3%) nodules exhibited hypoechogenicity in the solid part of the partially cystic nodules (**Figure 1**), and five (21.7%) nodules exhibited isoechogenicity in the solid part. Seventeen (73.9%) nodules had internal blood flow. For the CEUS parameters, 10 (43.5%) nodules exhibited hypoenhancement (**Figure 2**), and 22 (95.7%) nodules had heterogeneous enhancement (**Figure 2**), which meant that the microbubbles in the majority of the nodules were unevenly distributed. Twenty (87.0%) nodules had a centrifugal perfusion pattern, and three nodules (13.0%) had a centripetal perfusion pattern (**Figure 2**), indicating that most of the nodules received a perfusion of microbubbles from the center to the periphery. The quantitative CEUS parameters showed that 14 (60.1%) nodules had a PI index <1 (**Figure 2**), 14 (60.9%) nodules had a TP index ≥1, and 21 (91.3%) nodules had an AUC index <1 (**Figure 2**). In the benign thyroid nodule group, 15 (40.5%) nodules had a cystic percentage greater than 50%, 29 (78.4%) nodules had an absence of calcification, 15 (40.5%) nodules exhibited hypoechogenicity in the solid part, and 22 (59.5%) nodules exhibited isoechogenicity in the solid part. Twenty-three (62.2%) nodules had internal blood flow. For the CEUS parameters, 31 (83.8%) nodules exhibited hyperenhancement or isoenhancement, 30 (81.1%) nodules had uniform enhancement, and 34 (91.9%) nodules had a centripetal perfusion pattern. The quantitative CEUS parameters showed that 31 (83.8%) nodules had a PI index ≥1, 30 (81.1%) nodules had a TP index <1, and 28 (75.7%) nodules had an AUC index ≥1. The univariate analysis indicated that the PCPTCs more frequently presented with calcification, hypoechogenicity of the solid part, hypoenhancement, heterogeneous enhancement, centrifugal perfusion, PI index <1, TP index ≥1, and AUC index <1 for preoperative US and CEUS compared to benign thyroid nodules (all p < 0.05).

**Table 2:** Ultrasound characteristics of the PCPTCs and nodular goiters.

Characteristics	PCPTCs (n = 23)	Nodular goiter (n = 37)	p Value
**Conventional US parameters**			
Size (mm)	29.35 ± 12.21	32.97 ± 14.39	0.320
Cystic percentage			0.282
≥50%	6 (26.1)	15 (40.5)	
<50%	17 (73.9)	22 (59.5)	
Calcification			0.000*
Present	21 (91.3)	8 (21.6)	
Absent	2 (8.7)	29 (78.4)	
Solid part echogenicity			0.004*
Hypoechogenicity	18 (78.3)	15 (40.5)	
Isoechogenicity	5 (21.7)	22 (59.5)	
Internal vascularity			0.408
Present	17 (73.9)	23 (62.2)	
Absent	6 (26.1)	14 (37.8)	
**CEUS parameters**			
Enhancement type			
Hypo-	10 (43.5)	6 (16.2)	0.020*
Hyper- or iso-	13 (56.5)	31 (83.8)	
Enhancement uniformity			0.000*
Homogeneous	1 (4.3)	30 (81.1)	
Heterogeneous	22 (95.7)	7 (18.9)	
Perfusion			0.000*
Centripetal	3 (13.0)	34 (91.9)	
Centrifugal	20 (87.0)	3 (8.1)	
PI index			0.000*
≥1	9 (39.1)	31 (83.8)	
<1	14 (60.9)	6 (16.2)	
TP index			0.001*
≥1	14 (60.9)	7 (18.9)	
<1	9 (39.1)	30 (81.1)	
AUC index			0.000*
≥1	2 (8.7)	28 (75.7)	
<1	21 (91.3)	9 (24.3)	

*p < 0.05 was considered a significant difference.

PI, peak intensity; TP, time to peak; TP, time to peak time; AUC, area under the curve; PCPTC, partially cystic papillary thyroid carcinoma.

For evaluation of the value of CEUS parameters in PCPTCs, a binary logistic regression analysis was performed for all of the statistically significant CEUS variables (p < 0.05). The results indicated that enhancement uniformity (B = 4.080, OR = 59.166, 95% CI = 1.928–1,815.846, p = 0.020), centrifugal perfusion (B = 4.502, OR = 90.157, 95% CI = 4.443–1,829.637, p = 0.003), and PI index <1 (B = 5.515, OR = 248.279, 95% CI = 1.655–37241.707, p = 0.031) were independent characteristics related to the PCPTC nodules that could be used to differentiate them from benign nodular goiters (**Table 3**).

**Table 3:** Multivariate logistic regression analysis of CEUS characteristics related to PCPTCs distinguishing from nodular goiters.

Characteristics	Partial regression coefficient, β	Odds ratio	95% Confidence interval	p Value
Heterogeneous enhancement	4.080	59.166	1.928–1,815.846	0.020*
Centrifugal perfusion	4.502	90.157	4.443–1,829.637	0.003*
PI index <1	5.515	248.279	1.655–37,241.707	0.031*

*p < 0.05 was considered a significant difference.

PCPTC, partially cystic papillary thyroid carcinoma.

Heterogeneous enhancement, centrifugal perfusion, and PI index <1 were independent characteristics related to PCPTCs that could be used to differentiate them from benign thyroid nodes; therefore, we chose these CEUS parameters for the diagnosis of PCPTCs. If the thyroid nodule had more than 2 (≥2) of the above three CEUS characteristics, the thyroid nodule was classified as a malignant thyroid nodule; if the thyroid nodule had less than 2 (<2) of the above three CEUS characteristics, the thyroid nodule was classified as a benign thyroid nodule. Then, the combination diagnosis of Kwak TI-RADS classification and CEUS characteristics was calculated. If the thyroid nodule had a Kwak TI-RADS 4b score and/or CEUS ≥2, the thyroid nodule was classified as a malignant thyroid nodule; if the thyroid nodule had a Kwak TI-RADS 4a score and/or CEUS <2, the thyroid nodule was classified as a benign thyroid nodule. The diagnostic performance of the different methods for differentiation between benign and malignant thyroid nodules is outlined in **Table 4**. The Kwak TI-RADS with a cutoff value of 4a/4b score achieved an Az value of 0.851, with an accuracy of 86.7% (52/60). The CEUS characteristics with a cutoff value of CEUS ≥2 achieved an Az value of 0.924, with an accuracy of 91.7% (55/60). The combination of Kwak TI-RADS and CEUS achieved an Az value of 0.897, with a cutoff value of 4a/4b or CEUS ≥2, and had an accuracy of 88.3% (53/60), which was better than that of Kwak TI-RADS. However, there was no significant difference with respect to diagnostic accuracy for differentiation between benign and malignant thyroid nodules among all groups (p > 0.05).

**Table 4:** Diagnostic performance for discrimination between PCPTCs and nodular goiters.

Methods	Cutoff score	Sensitivity, %	Specificity, %	Accuracy, %	PPV, %	NPV, %	Az (95% CI)
TI-RADS	4a/4b	78.3	91.9	86.7	85.7	87.2	0.851 (0.738–0.963)
CEUS	CEUS ≥2	95.7	89.2	91.7	84.6	97.1	0.924 (0.848–1.000)
TI-RADS+CEUS	4a/4b or CEUS ≥2	95.7	83.8	88.3	78.6	96.9	0.897 (0.811–0.984)

TIRADS, Thyroid Imaging Reporting and Data System; CEUS, contrast-enhanced ultrasound; PPV, positive predictive value; NPV, negative predictive value; CI, confidence interval; PCPTC, partially cystic papillary thyroid carcinoma.

**Figure 3:**
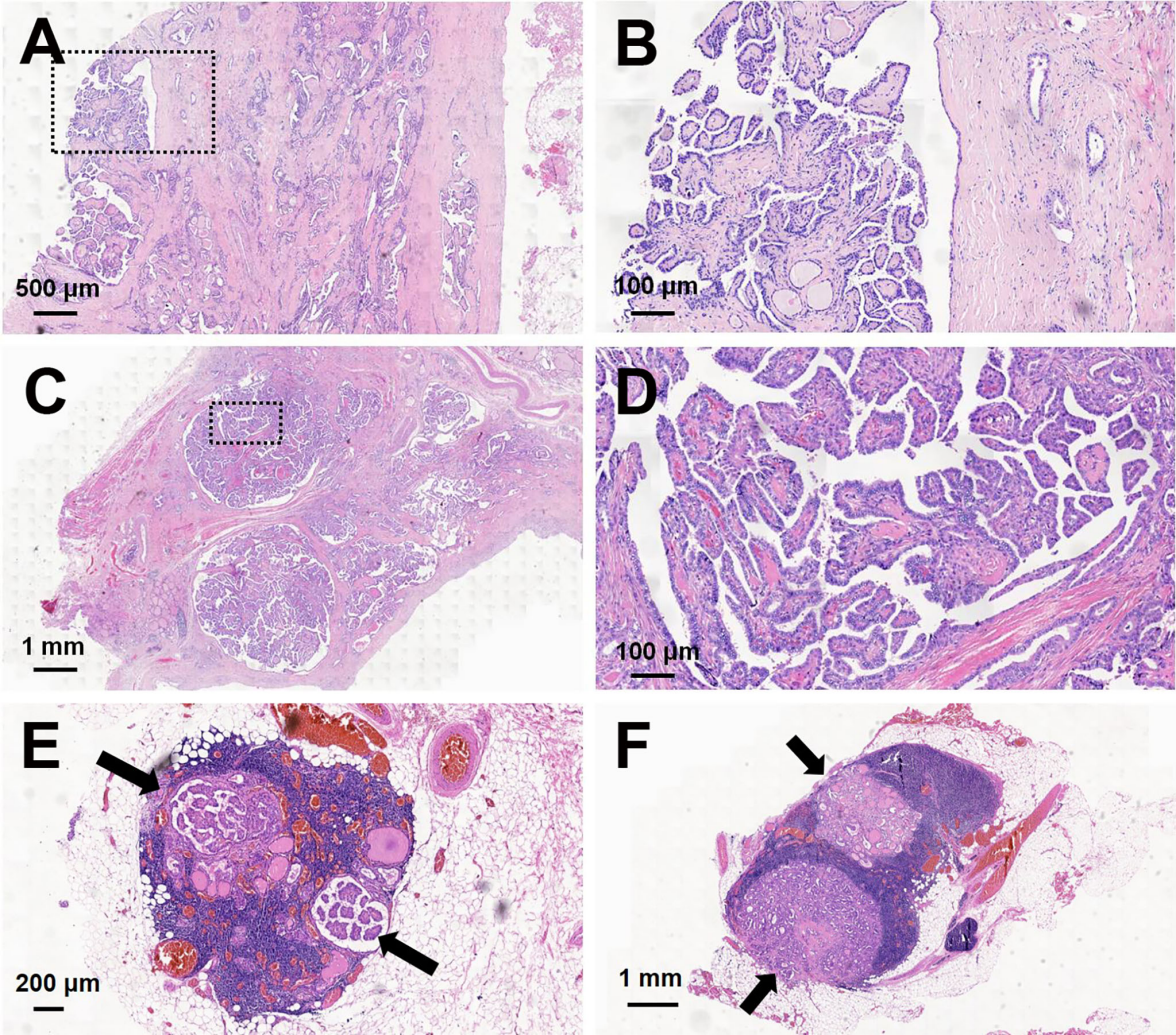
Hematoxylin and eosin (H&E) staining of predominantly cystic papillary thyroid carcinoma and central cervical lymph nodes. The thick cystic wall of the mass showed a lot of nipple-like bulges: **(A)** magnification, ×20; **(B)** magnification, ×100. The solid portion of the mass showed a lot of nipple-like bulges: **(C)** magnification, ×10; **(D)** magnification, ×100. Some nipple-like bulges (black arrows indicated) were shown in two central cervical lymph nodes: **(E)** magnification, ×25; **(F)** magnification, ×10.

Of 23 PCPTCs patients, 14 (60.1%) patients had central cervical lymph node metastasis. Histopathological examination of samples using hematoxylin and eosin (H&E) staining showed a predominantly PCPTC with a cystic percentage greater than 90% (**Figure 3**), revealing the thick cystic wall of the mass (3–6 mm) (**Figures 3A, B** **)** and the solid part abutting on the base side of the cyst wall (**Figures 3C, D**). Many nipple-like bulges were seen on both the cyst wall and the solid part. Meanwhile, some nipple-like bulges (black arrows indicated) were also observed in the central lymph nodes (**Figures 3E, F**), which indicated that those lymph nodes were metastatic for thyroid carcinoma.

## Discussion

Partially cystic thyroid nodules are common on ultrasonography and are considered to be a result of the cystic degeneration of either neoplastic or non-neoplastic nodules (11). Although partially cystic thyroid nodules are traditionally interpreted as having a low risk of malignancy, some studies have reported that the frequency of malignancy among partially cystic thyroid nodules varies from 5.2% to 17.6% (3, 6, 12, 13). A study by Kim et al. (14) compared the disease-free survival of 553 PTCs with cystic changes according to the percentage of cystic component (two groups: 25% or 50%) of the thyroid nodules and found that the proportion of the cystic component in PTCs did not affect disease-free survival. In this study, 56 patients (10.1%) were confirmed to have tumor recurrence within the follow-up period, while the independent predictors of recurrence were pathologic size, male sex, and lymph node metastasis. Therefore, it is vital to differentiate malignant thyroid nodules from benign partially cystic thyroid nodules using preoperative ultrasonography and/or US-FNAB.

Some studies have reported the conventional sonographic features of partially cystic thyroid nodules associated with malignancy. Lee et al. (12) reported that partially cystic thyroid nodules with a predominantly solid component (solid portion was >50%), an eccentrically placed solid component, and the presence of microcalcifications were all associated with malignancy. Kim et al. (3) reported that partially cystic thyroid nodules had an eccentric solid portion with an acute angle, and microcalcifications were significantly associated with malignancy. Park et al. (15) reported the malignant sonographic features of an entire partially cystic thyroid nodule and its internal solid portion. A taller-than-wide shape and spiculated or microlobulated margins were associated with malignancy in partially cystic thyroid nodules. Furthermore, an eccentric solid configuration, non-smooth margin, hypoechogenicity, and microcalcifications of the internal solid portion were significantly associated with malignancy. In our study, most PCPTCs had an eccentric solid portion with microcalcifications, which was consistent with recent reports (3, 12, 15). However, the acute angle of the internal solid portion, the taller-than-wide shape, and the spiculated or microlobulated margin of the entire nodule were not shown in our study (15). In a study by Henrichsen et al. (1), 360 malignant thyroid nodules that had been surgically removed were analyzed, nine (2.5%) of which were 51%–100% cystic. Of the nine malignancies (cystic portion >50%), four (44%) demonstrated increased vascularity either in a nodule or in thickened walls (1). In our study, some PCPTCs showed poor blood flow signals in the internal solid portion of the thyroid nodule, which was similar to the Color Doppler Flow Imaging (CDFI) findings of solid PTCs reported by other studies (10, 16). However, there are very few reports regarding the CEUS findings of malignant partially cystic thyroid nodules.

CEUS, as a novel technique for detecting microvessels of tissues, has been widely applied in improving the diagnostic accuracy of thyroid nodules. Deng et al. (17) reported that hypoenhancement on CEUS correlated highly with a malignant diagnosis of thyroid nodules (sensitivity: 82.1%, specificity: 84.9%, accuracy: 84.0%, PPV: 71.9%, and NPV: 91.0%). Ma et al. (18) found that heterogeneous enhancement on CEUS showed the best diagnostic performance for papillary thyroid microcarcinoma, with the highest PPV of 88.0%, an accuracy of 83.7%, and a relatively high specificity of 83.9%. However, mixed cystic nodules or almost cystic nodules (cystic portion >75%) were excluded from previous studies due to the demand for elastography (10, 17, 18). In our study, 43.5% of PCPTCs showed hypoenhancement, 95.7% of PCPTCs showed heterogeneous enhancement, and 87% of PCPTCs exhibited centrifugal perfusion, which is inconsistent with the CEUS enhancement of solid PTCs reported in previous studies (10, 19, 20). Compared with benign nodular goiters, PCPTCs more frequently had hypoenhancement, heterogeneous enhancement, centrifugal perfusion, PI index <1, TP index ≥1, and AUC index <1 on preoperative CEUS. Binary logistic regression analysis demonstrated that heterogeneous enhancement, centrifugal perfusion, and PI index <1 are independent CEUS characteristics related to malignant PCPTCs that can be used to differentiate them from benign nodular goiters (all p < 0.05). This may be due to increased neovascularization in the malignant nodules; however, the invasive growth characteristics of malignant nodules also destroyed the new blood vessels, formed small tumor thrombi in the necrotic vessels, and led to the occlusion of small blood vessels. Therefore, the degree of CEUS enhancement not only is related to the number of blood vessels but also depends on the state of internal blood vessel function. Especially in PCPTCs with cystic percentage greater than 90%, the solid portion on the cyst wall of the thyroid nodule had few blood vessels around the papillary protrusion in the cyst wall based on the H&E histopathological staining results, which was consistent with the heterogeneous hypoenhancement on CEUS. These results may be explained by the fact that the inner solid portion of the malignant nodule was often located at the base of the papillary protrusion in the cyst wall, and the blood vessels around the lesion easily invaded outward, resulting in ischemic necrosis of the inner solid portion near the side of the cystic portion and continuous cystic degeneration. Furthermore, the inner solid portion of the malignant nodule was located at the base of the papillary protrusion in the cyst wall, and the blood vessels around the lesion easily invaded outward, which may lead to central cervical lymph node metastasis.

This study had several limitations. First, an unavoidable selection bias may have existed, and some patients with suspicious malignancies might not have been enrolled in this study because they did not have surgery. Second, the nodule sizes studied for comparison between the two groups ranged from a few millimeters to tens of millimeters; these size differences might affect the US characteristics of PCPTCs. Third, all conventional US and CEUS examinations were performed by a single experienced operator, which means that the study was not operator independent. Because subjective interference caused by other examiners might exist, further studies with more operators performing each examination are needed. Fourth, this was a small-scale retrospective study, which may have caused the inaccurate evaluation of the effect on CEUS parameters by the different nodule sizes of the two groups. Thus, a large-scale prospective study is needed to clarify these findings in the future.

## Conclusion

PCPTCs are extremely rare cystic PTCs, and very few studies describe their ultrasonographic imaging features and related pathologic findings. On preoperative US and CEUS, PCPTCs more frequently presented with calcification, hypoechogenicity of the solid part, hypoenhancement, heterogeneous enhancement, centrifugal perfusion, PI index <1, TP index ≥1, and AUC index <1 compared with benign nodular goiters. Binary logistic regression analysis demonstrated that heterogeneous enhancement, centrifugal perfusion, and PI index <1 are independent CEUS characteristics related to malignant PCPTCs that can be used to differentiate them from benign nodular goiters. Thus, preoperative CEUS characteristics may serve as a useful tool to distinguish malignant PCPTCs from benign thyroid nodules and thus effectively supplement conventional US.

## Data Availability Statement

The raw data supporting the conclusions of this article will be made available by the authors without undue reservation.

## Ethics Statement

The studies involving human participants were reviewed and approved by the Ethical Committee of the Second Xiangya Hospital, Central South University. The patients/participants provided their written informed consent to participate in this study. Written informed consent was obtained from the individual(s) for the publication of any potentially identifiable images or data included in this article.

## Author Contributions

CN contributed to the conception and design of the work. FF and YG participated in the data analysis and article writing. LL, FY, ZZ, ZQ, and XL participated in the data collection and patients’ follow-up. All authors contributed to the article and approved the submitted version.

## Funding

This project was funded by the National Natural Science Foundation of China (81974267) and the Science and Technology Innovation Program of Hunan Province (2021RC3033).

## Conflict of Interest

The authors declare that the research was conducted in the absence of any commercial or financial relationships that could be construed as a potential conflict of interest.

## Publisher’s Note

All claims expressed in this article are solely those of the authors and do not necessarily represent those of their affiliated organizations, or those of the publisher, the editors and the reviewers. Any product that may be evaluated in this article, or claim that may be made by its manufacturer, is not guaranteed or endorsed by the publisher.
